# Influence of PKG on insulin signalling and GSK3 phosphorylation in SH-SY5Y cells

**DOI:** 10.1186/2050-6511-14-S1-P60

**Published:** 2013-08-29

**Authors:** Abhishek Sanyal, Didier Lochmatter, Anne Preußner, Alexander Pfeifer

**Affiliations:** 1 Institute for Pharmacology and Toxicology, University of Bonn, 53105 Bonn, Germany

## Background

Extracellular Amyloid-β (Aβ) plaques and intracellular neuro-fibrillary tangles (NFTs) of hyperphosphorylated Tau (τ) protein are considered to be the hallmarks of Alzheimer’s disease (AD) [1]. Aβ is secreted due to the sequential cleavage of the amyloidβ precursor protein (APP) by β- and γ- secretases (β cleavage) [1], whereas the intracellular signalling protein glycogen synthase kinase 3 (GSK3) has been implicated to cause τ- hyperphosphorylation leading to the formation of NFTs [2].

It has been shown earlier that the cleavage of APP by α- and γ- secretases (α- cleavage) is enhanced by insulin through the PI3K- Akt pathway [3]. GSK3 is a further downstream component of this pathway which has been shown to induce τ phosphorylation. Inhibition of GSK3 has also been shown to increase lysosomal biogenesis leading to autophagic degradation of APP [4].

## Materials and methods

For the purposes of this study, human neuroblastoma (SH-SY5Y) cells were used. Cells expressing wtAPP, bovine cGMP dependent protein kinase 1-alpha (PKG1α) and murine PKG2 were generated by lentiviral transduction and were stimulated with 200µM 8-pCPT-cGMP or/and 1µM insulin for 15 min or 2 hrs. The cells were then lysed and the proteins analysed by Western Blotting.

## Results

SH-SY5Y cells stably overexpressing APP, PKG1α and PKG2 were used to analyze the crosstalk between the cGMP-PKG and insulin signalling cascades that was reported in brown adipose tissue [5]. As is known, upon insulin stimulation, the APP overexpressing cells showed a marked increase in the α-cleavage of APP with increased secreted APPα levels (sAPPα). Analyzing the insulin pathway components in the cells overexpressing PKG (1α or 2), a significant increase in phosphorylation of GSK3 was also seen when these cells were stimulated with cGMP, implying that PKG influences the downstream phosphorylation events in insulin signalling. While, PKG1α overexpressing cells also showed a marked reduction in intracellular holoAPP levels with consequent reduction in extracellular sAPPα levels as well.

## Conclusion

Our results suggest a possible crosstalk between cGMP-PKG and insulin signalling cascades. PKG1α and PKG2 enhanced GSK3 phosphorylation upon cGMP stimulation, while PKG1α affected levels of intracellular holoAPP thus reducing extracellular sAPPα levels.

Influence of PKG on GSK3 phosphorylation renders it as a viable and valuable target for AD therapeutics following a two-pronged approach; to reduce secreted Aβ levels by enhancing lysosomal biogenesis and simultaneous τ hyperphosphorylation reduction.
